# Development and psychometric properties of a brief generic cancer knowledge scale for patients (BCKS-10)

**DOI:** 10.1007/s10552-022-01601-x

**Published:** 2022-07-12

**Authors:** J. Klein, C. Kofahl, E. Ziegler

**Affiliations:** Institute of Medical Sociology, University Medical Center Hamburg-Eppendorf, Martinistraße 52, 20246 Hamburg, Germany

**Keywords:** Cancer knowledge, Health literacy, Psychometrics, Patient education

## Abstract

**Purpose:**

This study aims to introduce the development and psychometric properties of a brief generic cancer knowledge scale for patients (BCKS-10) that includes different elements of knowledge and skills (terminology, diagnosis, treatment, prevention, and numeracy). Although cancer knowledge is a central dimension of cancer literacy, most previous studies either investigated cancer knowledge among the general population, or among patients with a specific cancer diagnosis.

**Methods:**

Qualitative interviews (*n* = 11) and a quantitative survey (*n* = 267) among peer support group leaders were conducted to further develop the BCKS-10 after literature screening. *n* = 500 patients with cancer were recruited across Germany between October 2020 and February 2021. Construct validity, item discrimination and reliability were tested.

**Results:**

ANOVA revealed no significant differences of the knowledge score between various cancer sites, a significant positive association with education, and a negative association with migration background by trend supporting construct validity. In terms of item discrimination, the corrected item-total correlation of 8 out of 10 items were above the threshold of 0.3. Cronbach’s alpha of about 0.68 revealed an acceptable internal consistency as the tool is brief and consists of different dimensions.

**Conclusion:**

Overall, the findings show that the BCKS-10 is a suitable tool to briefly assess cancer knowledge among patients independent of cancer site. However, further surveys have to be conducted to validate the psychometric properties and enhance the BCKS-10.

**Supplementary Information:**

The online version contains supplementary material available at 10.1007/s10552-022-01601-x.

## Introduction

During the past decades, health literacy became a highly relevant factor of health management and an important topic of patient-centred care [1, 2]. Reviewing the various definitions and conceptual models of health literacy, knowledge is an essential dimension when examining health literacy [3]. An overview of existing measurement tools of health literacy found a trend towards a mixed measurement including self-perceived and objective tests, as the assessment of objective knowledge widens the possibility to address multiple skills (e.g. numeracy) [4]. However, the study reports psychometric weaknesses of current tools and highlights the need to develop further instruments. These findings also hold true for cancer literacy. In terms of complex chronic diseases like cancer and a more and more challenging navigation within the health care systems, cancer knowledge is a substantial component of patients’ health, well-being and patient safety [5]. One study showed that health literacy, assessed by an objective knowledge test, is an independent predictor of cancer patients’ hospitalizations in the first five years after their diagnosis [6]. Moreover, a current overview summarised that lower health literacy was associated with greater difficulties to understand and process cancer related information as well as poorer quality of life and poorer experience of care [7]. Following a systematic review from 2015 [8], there is a limited number of cancer literacy and/or cancer knowledge measurement tools, a lack of reporting psychometric properties and no established inventory to date. Further, numeracy is a substantial dimension of health literacy [1, 9]. Numbers and numeric-based concepts are highly relevant regarding health-related communication and decision making. In terms of cancer, the assessment of risks, odds and prognoses as wells as the understanding of drug leaflets and further health information are essential skills for patients. Previous studies that assessed cancer knowledge either investigated knowledge among the general population or among patients with a specific cancer diagnosis [10–15]. Surveys among the general population are also relevant (e.g. in terms of early detection). However, cancer patients need particular knowledge about treatment options, medical terms and definitions [5]. Furthermore, many studies defined knowledge solely relating to symptom interpretation and not as a multidimensional construct [16]. Only one study examined generic cancer knowledge among patients regardless of cancer site, reporting good psychometric properties and limited cancer knowledge in about 18% [17]. The rationale for BCKS-10 was to develop a cancer knowledge scale, which (1) is brief, (2) refers to cancer patients (and not to the general population/laypersons), (3) is applicable for various cancer diagnoses, and (4) includes several dimensions of knowledge (i.e. terminology, diagnosis, treatment, prevention, legal matters and numeracy). The aim of this paper is to introduce the development of the BCKS-10, its components and psychometric properties.

## Methods

### Instrument development and design

There were three phases to the development process of the BCKS-10: (1) screening literature for existing instruments, (2) qualitative expert interviews with cancer peer support group leaders, (3) quantitative survey of cancer peer support group leaders. Out of the existing literature, particularly the Numeracy Understanding in Medicine Instrument (NUMi) (*n* = 1000 (general population); Cronbach’s *α* = 0.86) [9], and the Test for Ability to Interpret Medical Information (TAIMI) (*n* = 6047 (general population); Cronbach’s *α* = 0.36–0.51) [18] were considered for further development of the BCKS-10 in terms of numeracy skills. To include the patients’ perspective and needs into the development process, expert interviews (*n* = 11) and a survey (*n* = 267), both among cancer peer group leaders, were conducted. Both investigations were focused on the patients’ health care situation as well as current gaps and requirements of cancer knowledge, comprehension and communication. The BCKS-10 is part of a 13 items scale. The items 11–13, however, are nation-specific as they refer to knowledge about legal matters and administration in Germany, and thus, they are not appropriate for international use. Although data from the interviews and survey showed that this topic is relevant, these items were excluded from the international version due to large differences in the health care systems and legal conditions (i.e. items concerning the beginning of rehabilitation, the application of a disabled person’s card, and the duration of sick pay). Therefore, only the first 10 items were included in the present analysis. Based on the experts’ interviews and survey data, eight items regarding terminology, diagnosis, treatment and prevention were originally developed. As the interpretation of medical data was also mentioned as important for patients, two numeracy and data interpretation items (no. 3 and 5 of the BCKS-10) were adopted from the original NUMi (question no. 10) and TAIMI scale (question no. 5) and included in the instrument [9, 18] (see Online Resource for the complete instrument). The score ranges from 0 to 10 and a higher score indicates a higher cancer knowledge. A short pre-test (*n* = 13) was conducted in two hospitals in Hamburg. The results were assessed in collaboration with experts and clinicians and were rated as sufficient.

### Participants and setting

For recruitment, a multi-channel approach was applied to reach a large variety of cancer patients in different phases of treatment and areas of health care. Throughout Germany, the following organisations, facilities and institutes were contacted: hospitals with an oncological ward, rehabilitation clinics for cancer patients, cancer counselling centres, cancer societies of the federal states, comprehensive cancer centres, oncological practices as well as self-help organisations and self-help groups (via snowball sampling). Additionally, the study was advertised via public relations. Eligible participants were adults (18 ≥ years) and diagnosed with cancer regardless of cancer site, stage or time of the diagnosis. The study participation was voluntary and anonymous. Patients could participate online or alternatively via paper–pencil questionnaire. The study is part of the research project “Health literacy, self-help activities and health care experience of people with cancer’’ (gesa-K). The BCKS-10 is part of a larger multidimensional comprehensive survey that, in addition to the BCKS-10, includes tools to assess health status, health care experiences, health-related quality of life, coping, self-help activity and sociodemographic characteristics (www.uke.de/gesa-k). The survey commenced in October 2020, and the first 500 respondents that fully completed the BCKS-10 questionnaire were included in the psychometric analysis.

### Analyses

The psychometric properties of the BCKS-10 were tested in various ways. Based on previous research, construct validity was assessed by testing assumptions about expected differences in knowledge scores among the sample [19]. A positive correlation of the BCKS-10 score with the educational level (years of schooling) of the respondents, and a negative correlation with the migration status (no = 0; yes = 1) were assumed (a person has a migration background, if he/she or one of his/her parents were born abroad) [9, 16, 17]. Furthermore, no significant differences regarding the individual cancer site were presumed as the tool was aimed to be generic and not to be focused on a specific cancer site. Analyses of variance (ANOVA) were conducted to test these conditions. For the latter, the five most frequent cancer sites in the sample were introduced in the analyses.

To evaluate reliability, the internal consistency was measured using Cronbach’s alpha. A common threshold for alpha is 0.7 [19], but should not exceed 0.9 as it indicates unnecessary redundancy [20]. For further item analysis, an item difficulty index ranging from 0 to 1 (high difficulty scores indicate a greater proportion of the sample who answered the question correctly), and the corrected item-total correlation (range from 0 to 1) to show the coherence between an item and all other items in the scale. Adjusted item-total correlations below 0.3 are not desirable [19]. Additionally, the mean score of the BCKS-10, standard deviation, median, skewness, kurtosis and Shapiro–Wilk-test on normality of distributions were calculated. All analyses were carried out using the Statistical Package for the Social Sciences (SPSS) 26 [21].

## Results

The sample characteristics are shown in Table 1. About 55% of the respondents were male, the mean age was about 63 years, ranging from 20 to 86 years. More than half of the respondents had a high or very high educational level and 8% a migration background. On average, six years passed since the time of the first cancer diagnosis. All UICC tumour stages (0–4) were represented. The five most prevalent cancer sites among the sample were prostate, breast, bladder, colorectum, and the subgroup including leukaemia, lymphoma and myeloma. Due to the recruitment processes nearly 62% of the patients previously or currently participated in peer support groups at the time of recruitment.

**Table 1:** Sample characteristics and distribution of variables (*n* = 500): *n* (%) or mean ± standard deviation

Sex *(0)*	
Female	228 (45.6)
Male	272 (54.4)
Age (years) *(3)*	62.9 ± 12.1
Education (years) *(0)*	
Low (≤ 9)	49 (9.8)
Middle (10)	133 (26.6)
High (12–13)	88 (17.6)
Very high (> 13/university degree)	230 (46.0)
Migration background *(3)*	
Yes	41 (8.2)
No	456 (91.2)
Time since diagnosis (years) (0)	5.9 ± 6.2
Tumour stage (UICC) *(11)*	
0	14 (2.8)
I	50 (10.0)
II	77 (15.4)
III	105 (21.0)
IV	36 (7.2)
Unknown	207 (41.4)
Cancer site *(0)*	
Prostate	139 (27.4)
Breast	125 (25.0)
Bladder	65 (13.0)
Colorectum	37 (7.4)
Leukaemia, lymphoma and myeloma	37 (7.4)
Head and neck	22 (4.4)
Thyroid	16 (3.2)
Ovary and peritoneum	11 (2.2)
Lung	8 (1.6)
Skin melanoma	7 (1.4)
Other	20 (4.0)
Multiple entities	13 (2.6)
Participation in peer support groups *(0)*	
No	191 (38.2)
Yes (current or former)	309 (61.8)

Number of missing data in italics

**Fig. 1:**
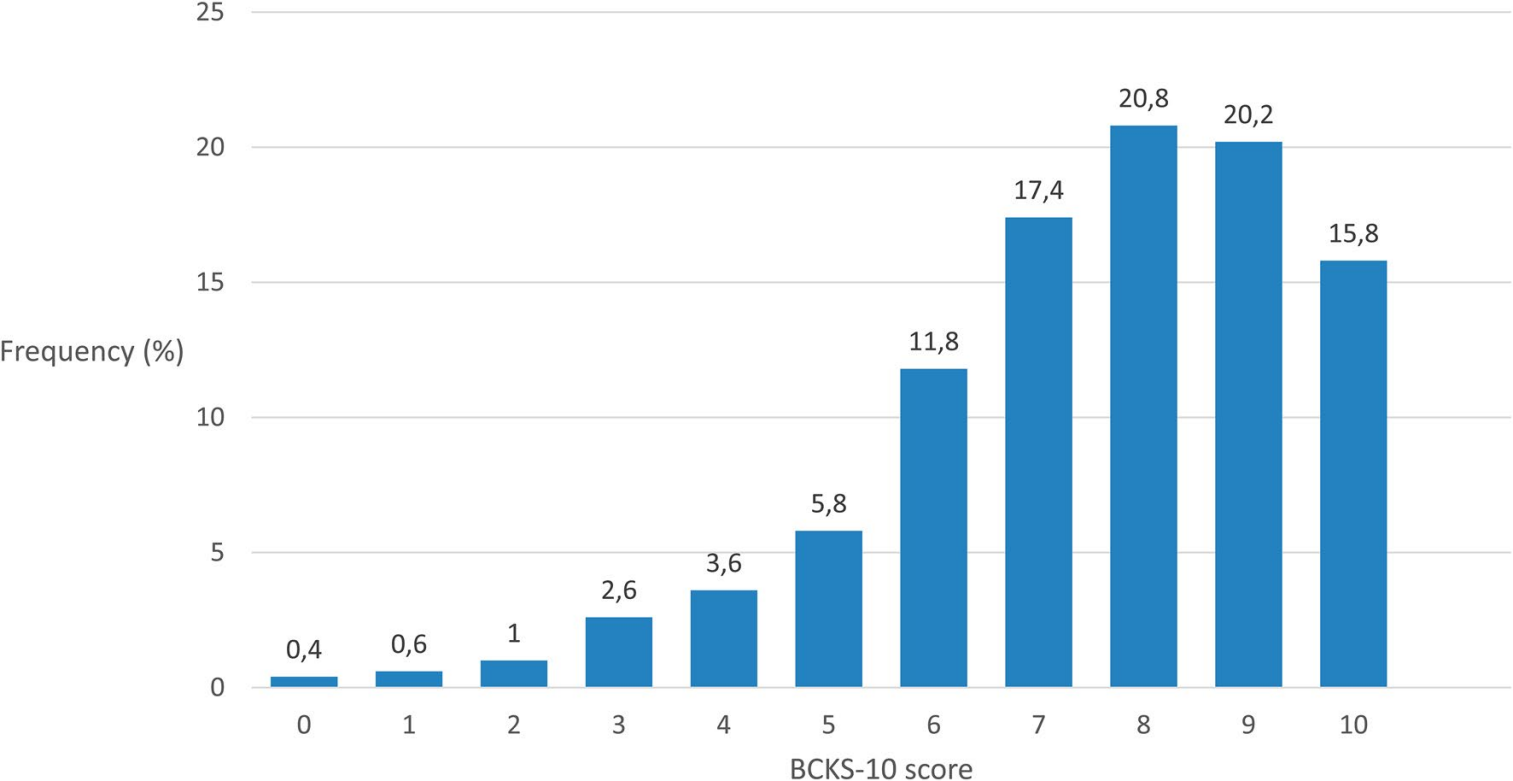
Distribution (%) of the BCKS-10 score (*N* = 500)

Figure 1 and Table 2 provide more information about the distribution and characteristics of the BCKS-10. The BCKS-10 score ranges from 0 (very low cancer knowledge) to 10 (very high cancer knowledge). The mean score was 7.53 (standard deviation 1.98). The distribution is left-skewed, however, the Shapiro–Wilk-test revealed a normal distribution.

**Table 2:** Distribution characteristics and internal consistency of the BCKS-10

n	M	SD	Mdn	Range	Skewness	Kurtosis	Shapiro–Wilk-test^a^	Cronbach’s α
500	7.53	1.98	8	0–10	− 0,954	0.901	0.140	0.680

*M* mean, *SD* standard deviation, *Mdn* median

^a^Test on normality of distributions

Three univariate ANOVA were calculated to test conditions for construct validity (Table 3). The mean scores for the BCKS-10 did not differ between patients of different cancer sites (*p* = 0.288). Furthermore, the analyses revealed highly significant differences between the educational groups indicating a clear gradient in favour of higher educated patients (*p* ≤ 0.001). In terms of migration status, the ANOVA did not show a significant difference (*p* = 0.193) but a trend towards a higher score among people without migration background. Multivariate analyses that were adjusted for education did not change significantly the results. Analyses of item difficulty and item discrimination (both with an overall range from 0 to 1) are presented in Table 4. The scores for item difficulty range from 0.432 to 0.990, the item discrimination varies from 0.244 to 0.414. In terms of reliability, the calculation of internal consistency showed a Cronbach’s alpha value of 0.680 (Table 2). Deleting items resulted in no improvements in Cronbach’s alpha value.

**Table 3:** BCKS-10 score (range 0–10), cancer site, education and migration background

	M ± SD	p
Cancer site		
Prostate	7.44 ± 2.17	0.288
Breast	7.71 ± 1.94	
Bladder	7.85 ± 1.85	
Colorectum	7.78 ± 1.67	
Leukaemia, lymphoma and myeloma	7.11 ± 1.66	
Education		
Low	6.10 ± 2.55	< 0.001
Middle	7.06 ± 1.97	
High	7.57 ± 1.68	
Very high	8.08 ± 1.98	
Migration background		
Yes	7.15 ± 2.55	0.193
No	7.57 ± 2.48	

Including the five most frequent cancer sites in the sample

*M* mean, *SD* standard deviation

*p* values are derived using univariate ANOVA

**Table 4:** Item difficulty and item discrimination of the BCKS-10 (*n* = 500)

Item	Item difficulty (range 0–1)	Item discrimination (corrected item-total correlation) (range 0–1)
1. A tumour stage I means	0.758	0.322
2. A drug is effective in 80% of those treated. That is, in how many people does it not work?	0.876	0.386
3. You have read that the incidence of adverse effects is 5%. What does that mean?	0.878	0.264
4. True or false? Palliative care aims to cure cancer	0.906	0.414
5. Rebecca was treated for breast cancer (stage II). There is a 10% chance that the cancer will come back in the next 10 years. If Rebecca takes a new drug, this probability is reduced by 30%. In how many out of 100 women taking the drug, like Rebecca, will the breast cancer come back in the next 10 years?	0.432	0.348
6. What is a metastasis?	0.990	0.244
7. What are cytostatics?	0.830	0.413
8. What is meant by a colonoscopy? An examination	0.832	0.336
9. Max utilize a cancer screening. This shows a conspicuous finding. However, the subsequent examination shows that Max does not have cancer. What is the term for such an early detection result?	0.522	0.384
10. What does the term “adjuvant therapy” mean?	0.502	0.396

## Discussion

This study aimed to introduce the development and psychometric properties of a brief general cancer knowledge scale for patients independent of cancer site and stage among a sample of cancer patients in Germany. Overall, the findings of the study provide evidence that the psychometric properties of the BCKS-10 are satisfactory. In previous literature, there is no consensus about the interpretation of Cronbach’s alpha values [22]. Mostly, a value ≥ 0.7 is rated as an acceptable threshold for reliability [19], but it is highly affected by the test length and dimensionality. The internal consistency in our study is about 0.68. Given the fact that scale only consists of 10 items, and that we introduce a knowledge scale that aims to test for discrete elements of knowledge and understanding in different dimensions and areas, the internal consistency can be seen as satisfactory [22]. In terms of item difficulty the range is from 0.43 to 0.99. Two items were correctly answered by more than 90% of the respondents. Upon the advice of the experts specifically from the patient organisations, we kept these items in the instrument, as solely items with a high difficulty potentially leads to frustration and dropout among the participants. Furthermore, no improvements in Cronbach’s alpha was achieved when deleting one of the items. Ceiling effects are considered to be present if more than 15% of respondents achieved the highest possible score [23]. In our study, 15.8% achieved a score of 10. Thus, a low ceiling effect cannot be ruled out. However, as higher educated patients are overrepresented in the sample, interpretations about the difficulty score should be done carefully. Further, an item-total correlation lower than 0.3 is not desirable [19]. In the present study, the values range from 0.24 to 0.41 including two items lower than the threshold. This could be seen as a limitation, however, the majority of the items showed an acceptable value. Construct validity is supported by the results regarding expected differences in knowledge scores among the sample. First of all, there is no difference in terms of cancer site. This is a relevant precondition as the instrument is meant to be generic for patients with cancer. In addition, the inclusion of interview and survey data among experts in the field highly contributed to the scale development and its validity. Furthermore, a clear and significant educational gradient was shown, as well as lower values among patients with migration background, although not significant. Concerning the participants with migration background, we have to add that these are supposed to be highly integrated and thus not representative for migrants in Germany as a whole, as they had a higher education, were following the request to participate in this study, and as they were in command of German language.

The BCKS-10 contributes to the current evidence of the assessment of cancer knowledge as there are only very few instruments that are brief, generic and addressed to cancer patients including different elements of knowledge and skills in terms of terminology, diagnosis, treatment, prevention and numeracy. While the validated CHLT-30 and the CLS were too long regarding the aim of a brief assessment, the also validated CHLT-6 lacks questions regarding treatment options and their terminology which was reported as relevant by the experts in our interviews and the survey [11, 17]. Further cancer knowledge tests lack data on reliability and validity, are based on specific cancer sites or solely assess reading abilities and numeracy [8, 24]. In the German version, three nation-specific items concerning legal matters of administration and health care system can be additionally included in the test, as facilitating of navigation within the health care system is one of the major recommendation of The German National Action Plan Health Literacy [25]. It is a suitable and convenient test that can be easily introduced in surveys among patients with cancer. The instrument is designed to quickly identify patients with limited and increased cancer literacy, and it allows to identify differences between subjective and objective measurements of knowledge and understanding of cancer in a survey.

Several limitations of the study have to be considered. Despite the multi-channel approach of participants’ recruitment, the sample cannot be regarded as representative for the whole collective of cancer patients in Germany. A selection bias cannot be ruled out as the sample predominantly consist of participants with a higher educational level and no migration background which may reduce the validity. Yet, low response rates among patients with lower education and migration background are common and still a relevant issue in survey research. The recruitment of patients with a chronic disease like cancer during the COVID-19 pandemic met numerous obstacles and diminished the quality of the sample. Therefore, the BCKS-10 should be applied and tested among further samples of cancer patients to confirm and potentially improve its psychometric properties. Additionally, solely the German version is tested in this study. However, the high percentage of previous or current members of peer support groups did not bias the results. An additional conduction of ANOVA revealed no significant differences in the BCKS-10 score between members and non-members (*p* = 0.237). Moreover, a further testing of construct validity by comparing the results with other established knowledge scales in a survey is recommended. Some values regarding the item difficulty and discrimination deviate from the common threshold which is already discussed above, and which also requires further surveys that include the BCKS-10 to improve the evidence. Nevertheless, our results suggest that the BCKS-10 is a suitable tool to briefly assess the knowledge of cancer among patients including different elements of knowledge. Instruments for cancer patients that are designed like the BCKS-10 are very rare and contribute to the investigation of cancer literacy, its education and improvement.

## Supplementary Information

Below is the link to the electronic supplementary material.

Supplementary file1 (PDF 536 kb)

## Data Availability

The datasets generated during and/or analysed during the current study are not publicly available due to protection of data privacy.
