# Supplementary material for: Development and psychometric properties of a brief generic cancer knowledge scale for patients (BCKS-10)
Source: Cancer Causes Control. 2022 Jul 12;33(9):1173–9. doi: 10.1007/s10552-022-01601-x (PMC9277979; doi:10.1007/s10552-022-01601-x)
Supplement: Supplementary file 1 — Supplementary file1 (PDF 536 kb) [file 10552_2022_1601_MOESM1_ESM.pdf]

Supplementary information

The BCKS-10 questionnaire (correct answers in bold)

1. A tumour in stage I means…

- **small or medium-sized tumours**
- tumours with metastases
- tumours with lymph node involvement
- tumours with distant metastases
- don't know

2. A drug is effective in 80% of those treated. That is, in how many people does it *not* work?

- 80 out of 100
- **20 out of 100**
- 8 out of 100
- 2 out of 100
- don't know

3. You have read that the incidence of adverse events is 5%. What does that mean?

- The majority of people will experience the adverse event.
- **There is a chance that an adverse event will occur in 5 out of 100 people.**
- An adverse event will occur during 5 out of 100 days.
- The severity of the adverse event is 5%.
- don't know

4. True or false? Palliative care aims to cure cancer.

- true
- **false**
- don't know

5. Rebecca was treated for breast cancer (stage II). There is a 10% chance that the cancer will come back in the next 10 years. If Rebecca takes a new drug, this probability is reduced by 30%. In how many out of 100 women taking the drug, like Rebecca, will the breast cancer come back in the next 10 years?

- 3 out of 100 women
- **7 out of 100 women**
- 10 out of 100 women
- 30 out of 100 women
- don't know

6. What is a metastasis?

- surgical procedure
- scientific analysis
- **secondary malignant growth**
- drug
- don't know

7. What are cytostatics?

- surgical procedures
- benign tumours
- secondary malignant growth
- **tumour-active drugs**
- don't know

8. What is meant by a colonoscopy? An examination...

- of the stomach
- of the brain
- of the blood
- **of the intestine**
- don't know

9. Max goes for a cancer screening. This shows a conspicuous finding. However, the subsequent examination shows that Max does not have cancer. What is the term for such an early detection result?

- correct positive
- **false positive**
- correct negative
- false negative
- don't know

10. What does the term "adjuvant therapy" mean?

- a treatment before the actual intervention (e.g. surgery)
- a treatment that directly targets the cancer
- a pure chemotherapy without surgery
- **a further treatment after the primary therapy (e.g. after the removal of a tumour)**
- don't know
